# Misdiagnosis and mistreatment of transverse testicular ectopia-two case report and review of literature

**DOI:** 10.1097/MD.0000000000035850

**Published:** 2023-11-03

**Authors:** Bo Yang, Chang-Hu Xie, Yin-Quan Wang

**Affiliations:** a Department of hernia and abdominal wall surgery. Shanxi Bethune Hospital, Shanxi Academy of Medical Sciences, Tongji Shanxi Hospital, Third Hospital of Shanxi Medical University, Taiyuan, China; b Tongji Hospital, Tongji Medical College, Huazhong University of Science and Technology, Wuhan, China.

**Keywords:** case report, congenital malformation, inguinal hernia, laparoscopic surgery, Persistent Müllerian duct syndrome, transverse testicular ectopia

## Abstract

**Rationale:**

Transverse testicular ectopia (TTE) is a rare congenital malformation with a high rate of misdiagnosis and mistreatment before operation, which cannot be diagnosed even during operation due to lack of knowledge.

**Patient concerns:**

Two patients with ectopic testes who were misdiagnosed as right inguinal hernia for the first time and underwent surgery. The “ovary” and “testicle” like structures was seen in the right inguinal region during the first operation. After being transferred to our hospital for laparoscopic surgery, it was found that the left spermatic cord and testis were transversely transverted to the right, the left testis was fixed at the right inner ring, and agglomerated soft tissue could be seen in the right inguinal canal, which was suspected to be Muller tube.

**Diagnoses:**

Based on preoperative images and intraoperative findings, both cases were diagnosed with Transverse testicular ectopia (TTE). The postoperative pathology report for the second patient revealed the presence of an in situ spermatogenic cell tumor in the ectopic testis.

**Interventions:**

Preperitoneal tension-free repair of right inguinal hernia and resection of left cryptorchidism were performed on the 2 patients.

**Outcomes:**

Postoperative pathology of the first patient confirmed that the resected specimens contained tubal-like and uterine-like structures. The postoperative pathology of the second patient showed that the resected tissue consists of immature testis, epididymis, uterus and seminal vesicle glands, in which an in situ spermatogenic tumor could be seen in the testicular tissue. Postoperative diagnosis: left transversal testicular ectopia and right indirect inguinal hernia.

**Lessons:**

The clinical misdiagnosis and mistreatment rate of TTE is very high. Once the patients with cryptorchidism complicated with inguinal hernia are found in clinic, the possibility of the disease must be considered. For the patients whose cryptorchidism does not descend into the ipsilateral scrotum and it is difficult to diagnose, laparoscopy can be used for both diagnosis and treatment. If a patient has both inguinal hernia and cryptorchidism, it is crucial to rule out a diagnosis of TTE to prevent misdiagnosis and inappropriate treatment.

## 1. Introduction

Transverse testicular ectopia (TTE) is a special type of testicular ectopia that is often associated with Persistent Müllerian duct syndrome (PMDS). Both testicles may be located in the ipsilateral scrotal or inguinal region and are often associated with inguinal hernia and hydrocele of the testicular sheaths. Adult patients are usually characterized by inguinal hernia or infertility. Because the disease is rare and studies are limited, it can be challenging to detect it in its early stages, resulting in a high rate of misdiagnosis. Therefore, we recommend enhancing preoperative ultrasonography and magnetic resonance imaging for patients with cryptorchidism and inguinal hernia. For patients with difficulty in preoperative diagnosis, laparoscopy can help in diagnosis and treatment.

In this paper, we introduce 2 patients with TTE who was misdiagnosed as unilateral inguinal hernia and during the first operation, “ovary” and “testicle” like structures was observed in the inguinal region. Then transferred to our hospital, reoperation confirmed that the patient had a transversed ectopic unilateral testis with a right indirect inguinal hernia and hydrocele. The disease is clinically rare, and the misdiagnosis and mistreatment rate is extremely high. The purpose of this paper is to explore the clinical experience and improve the understanding of the disease.

## 2. Case presentation

### 2.1. Case 1

A 45-year-old male patient was admitted to hospital because a reversible lump in the right inguinal region was found 2 months ago. Right inguinal hernia and left cryptorchidism were diagnosed, and surgical treatment of inguinal hernia repair was performed. During the operation, there was a 2cm × 2cm “ovary”-like structure in the hernia sac. The obstetricians and gynecologists were asked for consultation during the operation, but the diagnosis could not be confirmed. No hernia repair and other special treatments were performed after that. The incision was closed after exploration. The patient had an incision infection in the groin area after the operation, and was discharged after dressing change for about 1 month. The patient was treated in our hospital 2 months after the incision healed. There was no obvious abnormality in the patient history of past illness, personal and family history.

**Figure 1.**
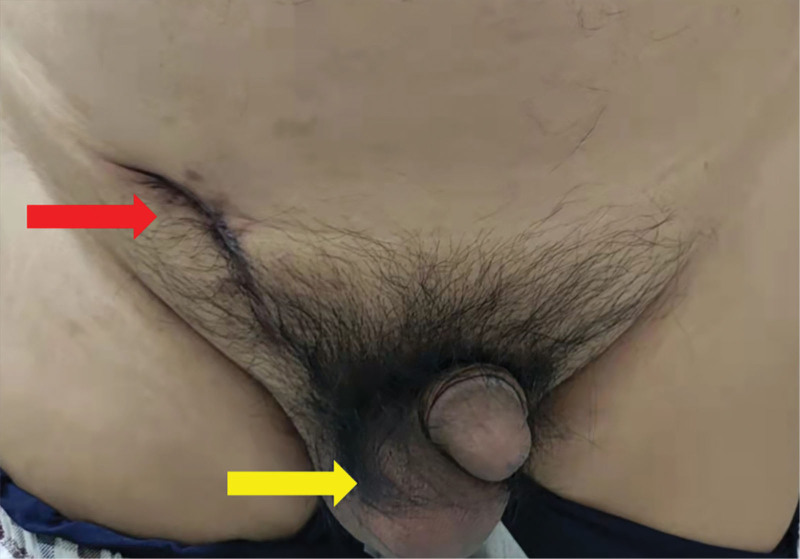
Clinical photograph before reoperation. Scar tissue in the right groin area (red arrow). The appearance of the right scrotum is normal, and the left scrotum is empty (yellow arrow).

**Figure 2.**
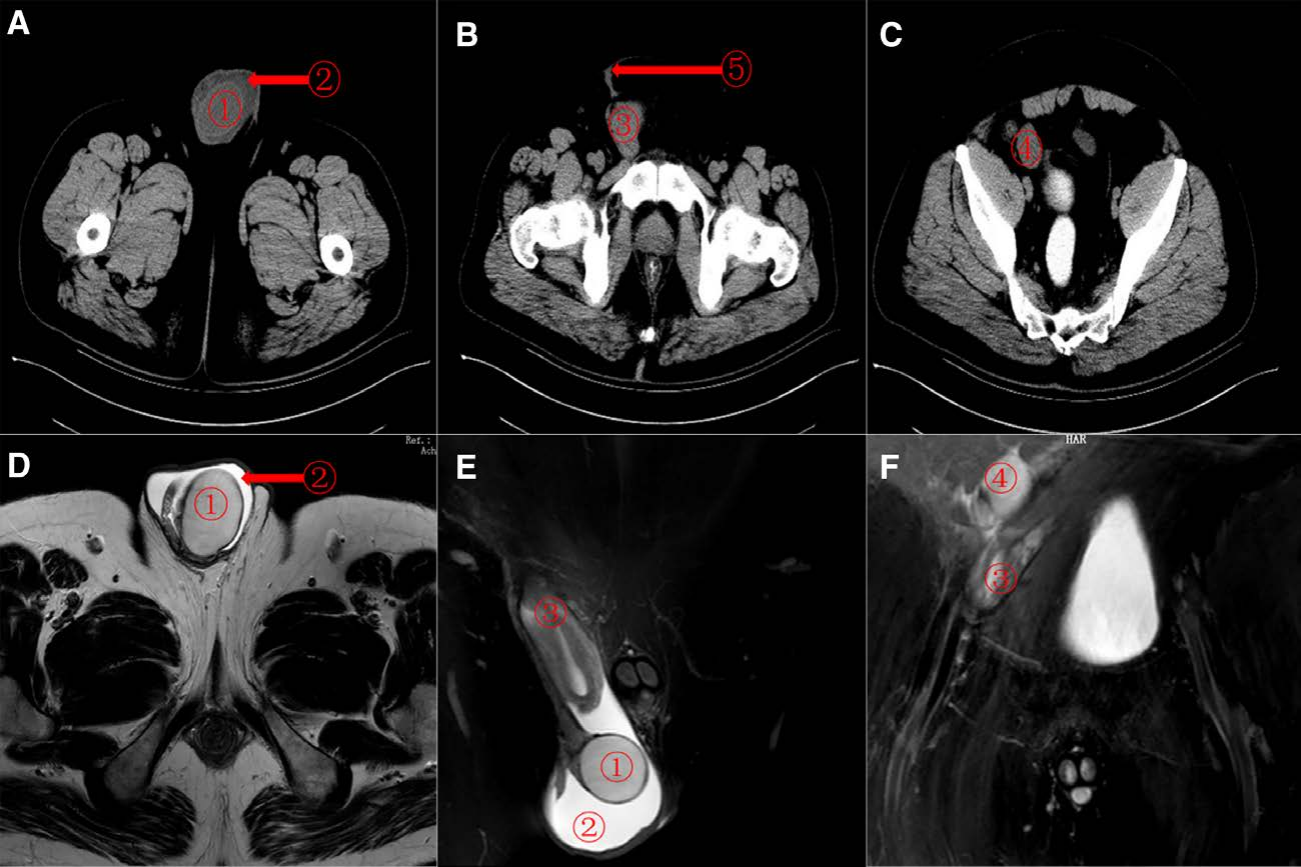
Preoperative CT (A–C) and MRI (D–F) images. ① Right testicle in normal position. ② Right testicular hydrocele. ③ Right inguinal canal tissue, mullerianduct tissue and left seminal vesicle gland? ④ Left ectopic testis in the right groin region. ⑤ Scar tissue in the right groin area. CT = computerized tomography, MRI = magnetic resonance imaging.

At physical examination, the left testis was not found, and the left inguinal region did not touch the testis and epididymis. Normal testes are palpable within the right scrotum, and scars are present in the right inguinal region. The scar tissue was hyperplastic, and the palpation was unsatisfactory. The right inguinal region does not touch testis and epididymis. The 5cm-sized mass could be seen in the right scrotum in the standing position, and the mass in the supine position could be reduced but could not disappear completely (Fig. 1). The scrotal light transmission test was positive. The patient laboratory examinations were normal. B-ultrasound examination showed a left scrotal void, no clear echo of the testis in the left inguinal region, and hydrocele of the right testes. Computerized tomography (CT) showed hydrocele in the right scrotal testis theca, cryptorchidism on the left side, absence of seminal vesicle gland on the left, abnormal soft tissue density in the right inguinal canal, and pulling the structure of the seminal vesicle gland on the left (Fig. 2 A–C). Magnetic resonance imaging (MRI) showed hemorrhage of the right capsule gland, absence of the left seminal vesicle gland, abnormal structure of the right inguinal region and postoperative changes of the right inguinal hernia (Fig. 2 D–F).

**Figure 3.**
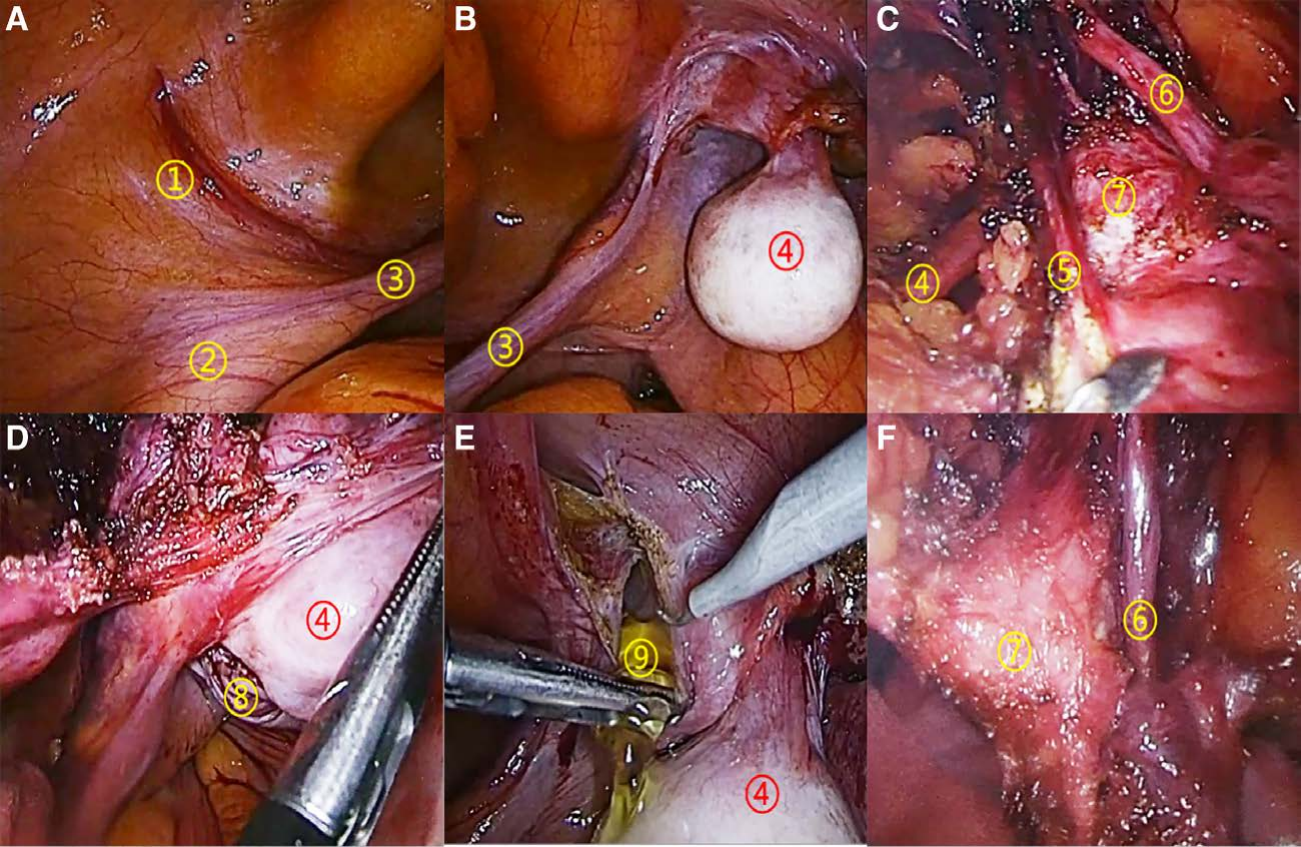
Diagnostic laparoscopy findings (A–F). ① Left reproductive vessel. ② Left vas deferens. ③ Left spermatic cord. ④ Left ectopic testis in the right groin region. ⑤ ⑥ ⑦ Mullerian duct tissue. ⑧ Right hernial sac. ⑨ Hydrocele of tunica vaginalis.

Laparoscopic surgery was performed. During the operation, it was found that the left spermatic cord and testis crossed ectopic to the right side, the left testis was fixed at the right internal ring, and the right spermatic cord can move when pulling the right testicle. The defect of the right inner ring is about 2cm × 2cm, and the soft tissue can be seen in the inguinal canal, which is suspected to be Muller canal structure. In addition, hydrocele of testis can be seen (Fig. 3). During the surgery, the right inguinal hernia was repaired using a Transabdominal preperitoneal (TAPP). The ectopic testicles located in the right inguinal region (which are normally located in the left scrotum) and the surrounding soft tissue were also removed during the operation.

Postoperative pathology confirmed that the resected specimen contained fallopian tube-like structure. The final diagnosis of the presented case was left transversal testicular ectopia and right indirect inguinal hernia, right testicular hydrocele, male pseudohermaphroditism. No postoperative complications was occurred. The patient was discharged on the fourth postoperative day. No recurrence of inguinal hernia was found in the follow-up for half a year.

### 2.2. Case 2

A 37-year-old male patient was admitted to hospital because a reversible lump in the right groin area was found 20 years ago. The patient was diagnosed with right inguinal hernia in a local hospital and underwent surgery 7 years ago. “Testicle” was found in the right hernia sac during surgery, and no hernia repair or other special treatment was performed. After exploration, the incision was closed. His younger brother had a right inguinal hernia and had a daughter. His past and personal history were unremarkable.

**Figure 4.**
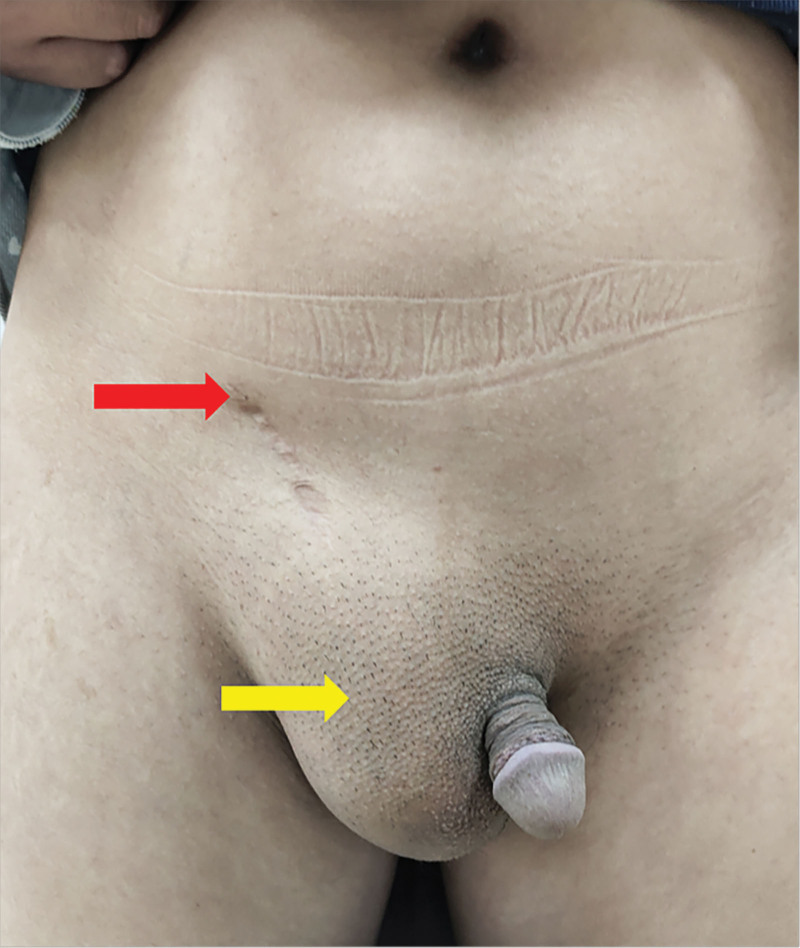
Clinical photograph before reoperation. Scar tissue in the right groin area (red arrow). The appearance of the right scrotum is normal, and the left scrotum is empty (yellow arrow).

**Figure 5.**
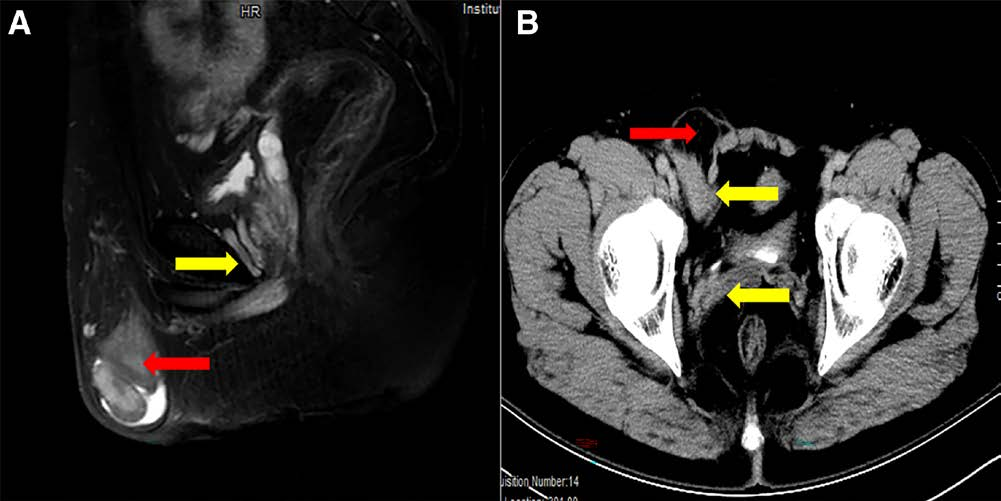
Preoperative MRI (A) and CT (B) images. Right hernia sac and tissue inside the hernia sac (red arrow). Abnormal “uterus” (yellow arrow). CT = computerized tomography, MRI = magnetic resonance imaging.

Physical examination on admission was almost the same as that of the first patient, with normal penile development, a left scrotal void, and no testes and epididymis were palpated in the left inguinal region. A reduced-volume testis was palpable within the right scrotum, and a mass approximately 1.5cm in size was palpable in the right inguinal region, suspicious of an ectopic left testis. The 5cm-sized mass could be seen in the right scrotum of the patient in the standing position, and the mass in the supine position could be reduced but could not disappear completely. The scrotal light transmission test was positive (Fig. 4). The patient laboratory examinations were normal. Ultrasound showed the size of the right testis was small, but no ectopic testis in the inguinal canal was seen. MRI examination showed that the size of the right testis was small, and the structure of the left testis and spermatic cord were not shown. CT showed a right inguinal hernia. Additionally, there was tissue resembling the uterus (Fig. 5 A and B).

**Figure 6.**
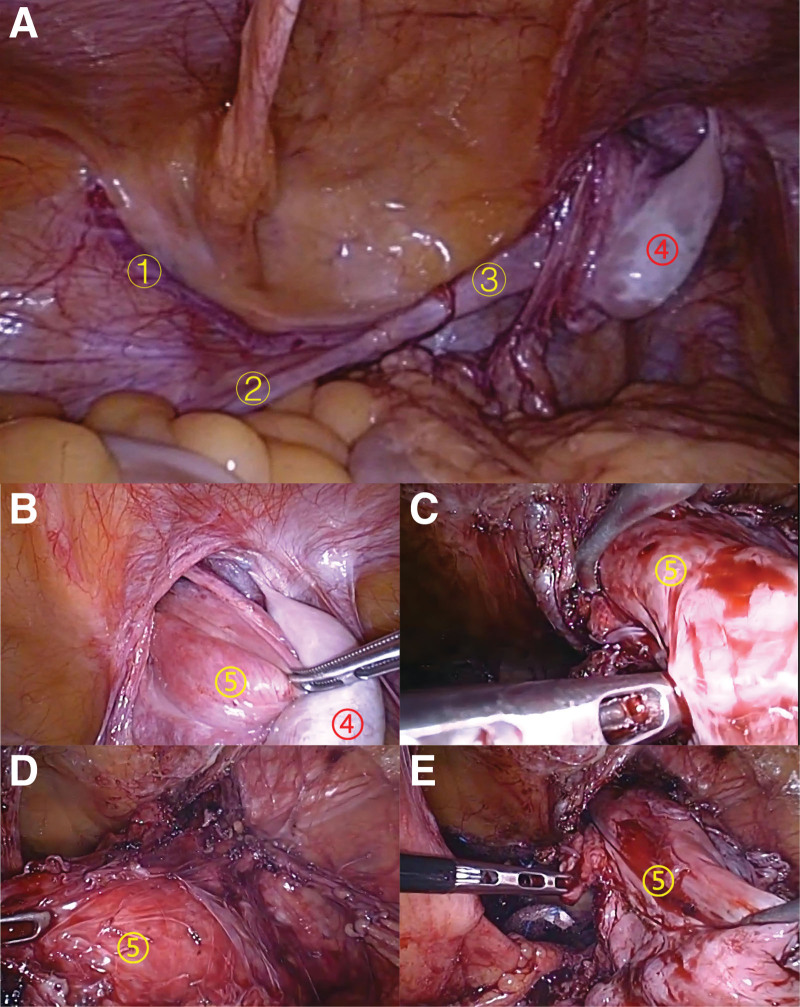
Diagnostic laparoscopy findings (A–E). ① Left reproductive vessel. ② Left vas deferens. ③ Left spermatic cord. ④ Left ectopic testis in the right groin region. ⑤ Abnormal “uterus.”

**Figure 7.**
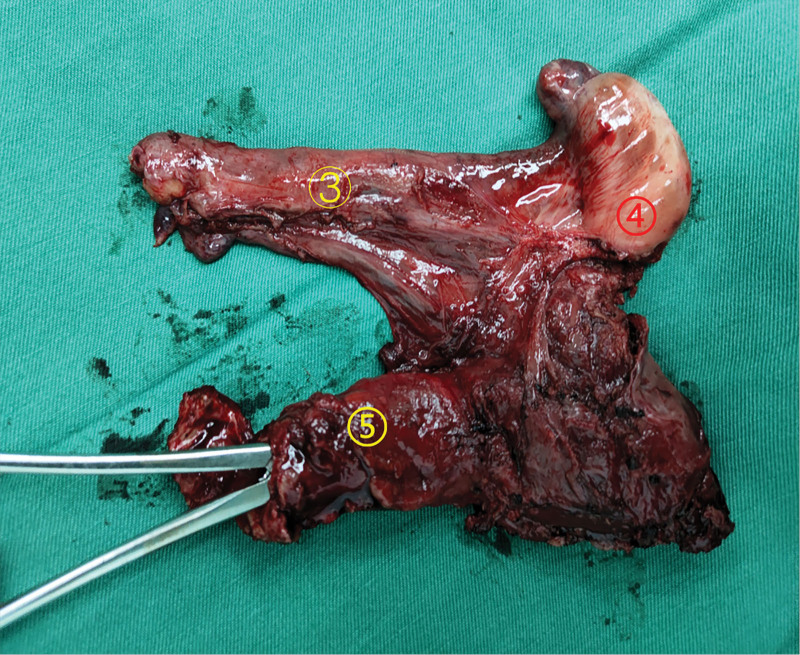
The specimen after laparoscopic surgery shown in Figure 6 above. (③ ④ ⑤ are the same as Fig. 6).

Laparoscopic surgical treatment was performed, and the intraoperative exploration findings were similar to those of the first patient, with the left spermatic cord and testis transversally ectopic to the right, except that the clear “uterus” structure was visible in the second patient (Figs. 6 and 7). During the operation, the surgeon repaired a right inguinal hernia and removed ectopic testicles that were located in the right inguinal region (which are normally located in the left scrotum). Additionally, uterine-like tissue in the right inguinal area was also removed. The final diagnosis of the case was left transversal testicular ectopia and right indirect inguinal hernia, right testicular hydrocele, male pseudohermaphroditism. The postoperative pathology showed that the resected tissue was composed of immature testis, epididymis, uterus and seminal vesicle glands, in which an in situ spermatogenic tumor could be seen in the testicular tissue. No postoperative complications was occurred. The patient was discharged on the fourth postoperative day. No recurrence of inguinal hernia was found in the follow-up for half a year.

## 3. Discussion

TTE is a rare congenital testicular dysplasia, the incidence of this anomaly is one in 4 million in children,^[1]^ in which the affected testis can be transferred to the contralateral side via the retroperitoneum or subcutaneously, and the bilateral testes can enter the ipsilateral scrotum or be fixed to the ipsilateral inguinal region or abdominal cavity. Patients may be complicated with permanent PMDS, inguinal hernia or hydrocele. Due to insufficient clinical understanding, they are often admitted to hospital with hydrocele, inguinal hernia or cryptorchidism.^[2]^ The rate of misdiagnosis and mistreatment is high. Most of the patients are infertile (neither of our 2 reported patients gave birth) , and the ectopic testicles of some patients can become malignant, which seriously affects the physical and mental health of the patients.^[3]^

There are many explanations for the causes of ectopic testes. The first explanation is that normal male testicles originate from different genitel ridge, and if 2 testes originate from the same genitel ridge, it may lead to ectopic testes. The second explanation is that Mullerian duct degeneration is incomplete, and its mechanical effect limits the normal decline of the testis, which eventually leads to testicular ectopia.^[4]^ The third explanation is that the abnormal development of the ipsilateral testicular gubernaculum may lead to TTE. The mechanism of the descent of the testis due to the gubernaculum is unknown, but the gubernaculum growth secondary to the enlargement of the scrotum is a reasonable explanation.^[5]^ The fourth explanation is that the secondary inguinal ring exists below the classic external inguinal ring, and the ring-like structure below it is called the secondary inguinal ring. If the abnormal development of this position leads to obstruction or atresia, the descent of the ipsilateral testis is blocked, and the testis can be descended through other possible channels, resulting in a variety of testicular ectopia including testicular transversal ectopia.^[6]^ In addition, some scholars believe that the development of bilateral vas deferens is normal if they originate from the same side, and it will lead to unilateral testicular ectopia normal if they originate from different sides.^[7]^ Some scholars believe that the testes descend from the same position to the bilateral scrotum, if there is a persistent abnormal connection between the testis and the vas deferens, or between the vas deferens and muller duct, it can eventually lead to TTE.^[8]^

TTE was generally divided into 3 types. The first type is unilateral cryptorchidism with contralateral indirect inguinal hernia. The second type is complicated with PMDS, and the hernia contents include visible uterus, fallopian tube and other tissues. The third type is combined with other malformations except PMDS, such as hypospadias, ureteropelvic junction obstruction and other urinary malformations.^[2,9]^ Previous statistics show that the first type is the most common, accounting for about 50% of the total medical records. However, recent reports show that more and more patients are combined with PMDS, accounting for about 49.6% and 35.3%, which may be related to the improvement of detection technology and the increase in the application of laparoscopic technology.^[10]^

The 2 patients we reported belong to type II and postoperative pathological examination confirmed the existence of Miller canal tissue in the resected tissue. This type of disease is even rarer clinically, Fewer than 270 well documented cases of TTE were reported by 2019.^[11]^ Most of them are case reports. PMDS is a special type of pseudohermaphroditism. The genotype of the patient is (46, XY) and the phenotype is male. In the early stage of embryonic development, both females and males have a pair of mesonephric ducts and a pair of Mullerian ducts. The mesonephric ducts are the primordia of the male reproductive system, and the Mullerian ducts are the primordia of the female reproductive system. In men, the androgen secreted by the Leydig cells promotes the development of the mesonephric duct into male reproductive duct, and the Mullerian duct inhibitor secreted by testicular Sertoli cells promotes the degeneration of Mullerian canal. In female, the mesonephric duct degenerates and mullerian duct develops into female reproductive duct. Mutations in AMH and AMHR2 genes may lead to deficiency of anti-mullerian hormone deficiency or reduced response of target organs to anti-mullerian hormone, resulting in the perpetuation of mullerian ducts and eventually lead to mullerian duct degenerative insufficiency syndrome.^[12,13]^

PMDS can be divided into 3 types. The first type is unilateral indirect inguinal hernia, with the contents of uterus and ipsilateral fallopian tube. The second type, in addition to the first-type features, involves the passage of the contralateral testicles and fallopian tubes from extraperitoneal across the midline into the inguinal hernial sac, known as testicular transversality. The third type, called the female type, presents with bilateral testes not palpable to the scrotum, with the uterus in the pelvis and bilateral testes embedded in the broad ligaments of the uterus, similar to the position of the ovary.^[14]^

The preoperative diagnosis rate of TTE is low.^[15]^ Most cases of TTE are diagnosed with inguinal hernia, hydrocele or infertility. Although the application of ultrasound CT and MRI in the diagnosis of TTE has improved the diagnosis rate, there is still a high rate of misdiagnosis and mistreatment.^[2,16]^ Ultrasonography is the most commonly used in the preliminary diagnosis and differential diagnosis of TTE, and is of great significance in guiding clinical treatment.^[17]^ It is easy to make a diagnosis in patients with bilateral testicles descending to the ipsilateral scrotum. However, due to the changeable position of the testis across the ectopic testis, there is still a certain risk of missed diagnosis of the testis located in the abdominal cavity or the upper inguinal canal, especially when the ultrasound physician is inexperienced. For patients with multiple operations, the specificity of ultrasonic diagnosis is reduced. Kullendorff et al reported that in 5 of the 8 patients who underwent reoperation, the surgical results did not match the preoperative ultrasound diagnosis.^[18]^ Kattak et al counted 11 children with reoperation and reached a similar conclusion.^[19]^ The 2 patients we reported were also reoperated, and preoperative ultrasound did not find the location of cryptorchidism, failed to make the diagnosis of TTE. MRI has relatively high sensitivity and specificity in locating testis, especially for patients who have undergone reoperation or multiple operations, and it has advantages when the operation area is sticky and the anatomical structure is mixed.^[16,20]^ However, because of the high cost of MRI and the need for longer examination time, TTE patients are mostly children, who can’t cooperate with the examination and often need to be sedated, so their use is limited.^[21]^ CT also plays an important role in the diagnosis of TTE,^[22]^ but it is not recommended in the diagnosis of TTE children because of its ionizing radiation.^[16]^ Laparoscopic technique can be used to diagnose and treat TTE, which is more and more widely used in clinic. It has a high accuracy in the diagnosis of TTE and cryptorchidism.^[23]^ For patients with difficulty in preoperative diagnosis, laparoscopic exploration can be performed during the operation to help further treatment.^[24]^

Testes of TTE patients are separated from the normal position and can be located in multiple positions such as the perineum or the abdominal cavity. The relatively high temperature can lead to infertility or even malignant. Statistics show that the rate of malignancy can be increased 5–10 times.^[21]^ Because of the poor effect of TTE on hormone therapy, surgical treatment is the only effective method. It is generally recommended that children aged from 6 to 18 months should complete the surgical treatment and fix the ectopic testis.^[25]^ For every 6 month increase in age at surgical treatment, the risk of malignant increased by 6%.^[26]^ So we recommended that surgical treatment should be performed promptly once diagnosed. Traditional surgical methods include fixation of ipsilateral testis after reduction, and fixation of testis across the scrotal mediastinum, etc.^[23]^ However, due to the rare occurrence and the common anatomic variations of the disease, the mullerian duct may be missed or the testicular blood supply may be damaged during the operation when the experience is insufficient. The application of laparoscopic technique can improve the therapeutic effect to some extent. During the operation, the type of TTE can be observed, whether PMDS is combined or not can be clarified, and the course of spermatic cord blood vessels on the affected side can be observed to guide the next surgical treatment.^[27–29]^

There is controversy about whether to resect mullerian duct during the operation. In the early reports, it was considered that there was no evidence for malignant transformation of the residual mullerian duct tissue, but instead the testicular blood vessels were damaged and the blood supply was affected during the resection. It was recommended that the resection be performed only when the mullerian duct tissue affected the testicular fixation.^[30,31]^ However, subsequent review by Farikullah et al showed that 11 cases of mullerian malignant tumor were found in about 200 cases, and the risk of mullerian malignant tumor for patients ranged from 3.1% to 8.4%.^[32]^ K Giri et al also reported a case in which mullerian duct tissue was preserved during surgery and advanced choriocarcinoma was diagnosed 25 years after surgery.^[33]^ Therefore, we believe that the course of the spermatic cord blood vessels should be clarified during the operation, so as to avoid damaging the testicular blood vessels and to resect the residual tissue of mullerian duct as much as possible.

Most patients with TTE present as children, however, in some areas, many patients are adults, and they often see a doctor with infertility, inguinal hernia or malignant tumor instead of TTE.^[3,34]^ For children over 10 years old and adults with TTE, orchiectomy on the affected side is generally advocated because the ectopic testis has been in high temperature environment for a long time and the risk of malignant transformation has increased significantly.^[35,36]^ Both of the patients we reported are adults. In case 2, ectopic testis was found during the operation, but surgical treatment was not performed in time, the patient had testicular cancer when he underwent reoperation 7 years later.

## 4. Conclusion

TTE is a rare hereditary disease. Due to insufficient understanding of this disease, the clinical misdiagnosis and mistreatment rate is extremely high. Once a patient with inguinal hernia complicated with cryptorchidism is found in clinical practice, the possibility of this disease must be considered. Preoperative ultrasound, magnetic resonance imaging and other examinations should be performed to confirm the diagnosis. For patients with cryptorchidism not descending into ipsilateral scrotum, preoperative diagnosis is difficult and laparoscopic has a certain advantage in the diagnosis and treatment.

## Acknowledgments

We sincerely thank the patient and his families for their participation and permission to publish this case report.

## Author contributions

**Conceptualization:** Bo Yang, Yin-Quan Wang.

**Data curation:** Bo Yang, Chang-Hu Xie.

**Methodology:** Bo Yang, Chang-Hu Xie, Yin-Quan Wang.

**Validation:** Bo Yang, Yin-Quan Wang.

**Writing – original draft:** Bo Yang.

**Writing – review & editing:** Bo Yang, Yin-Quan Wang.
